# WormBase 2017: molting into a new stage

**DOI:** 10.1093/nar/gkx998

**Published:** 2017-10-24

**Authors:** Raymond Y N Lee, Kevin L Howe, Todd W Harris, Valerio Arnaboldi, Scott Cain, Juancarlos Chan, Wen J Chen, Paul Davis, Sibyl Gao, Christian Grove, Ranjana Kishore, Hans-Michael Muller, Cecilia Nakamura, Paulo Nuin, Michael Paulini, Daniela Raciti, Faye Rodgers, Matt Russell, Gary Schindelman, Mary Ann Tuli, Kimberly Van Auken, Qinghua Wang, Gary Williams, Adam Wright, Karen Yook, Matthew Berriman, Paul Kersey, Tim Schedl, Lincoln Stein, Paul W Sternberg

**Affiliations:** Division of Biology and Biological Engineering 156–29, California Institute of Technology, Pasadena, CA 91125, USA; European Molecular Biology Laboratory, European Bioinformatics Institute, Wellcome Trust Genome Campus, Hinxton, Cambridge CB10 1SD, UK; Informatics and Bio-computing Platform, Ontario Institute for Cancer Research, Toronto, ON M5G0A3, Canada; Wellcome Trust Sanger Institute, Wellcome Trust Genome Campus, Hinxton, Cambridge CB10 1SA, UK; Department of Genetics, Washington University School of Medicine, St Louis, MO 63110, USA

## Abstract

WormBase (http://www.wormbase.org) is an important knowledge resource for biomedical researchers worldwide. To accommodate the ever increasing amount and complexity of research data, WormBase continues to advance its practices on data acquisition, curation and retrieval to most effectively deliver comprehensive knowledge about *Caenorhabditis elegans*, and genomic information about other nematodes and parasitic flatworms. Recent notable enhancements include user-directed submission of data, such as micropublication; genomic data curation and presentation, including additional genomes and JBrowse, respectively; new query tools, such as SimpleMine, Gene Enrichment Analysis; new data displays, such as the Person Lineage browser and the Summary of Ontology-based Annotations. Anticipating more rapid data growth ahead, WormBase continues the process of migrating to a cutting-edge database technology to achieve better stability, scalability, reproducibility and a faster response time. To better serve the broader research community, WormBase, with five other Model Organism Databases and The Gene Ontology project, have begun to collaborate formally as the Alliance of Genome Resources.

## INTRODUCTION

WormBase is a critically important public information resource for nematode research. There are three broad areas of research that WormBase targets: fundamental scientific research, biomedical research by studying model organisms and parasitic worm research. The first two areas are centered around the nematode species *Caenorhabditis elegans* and other experimentally tractable nematodes such as *Caenorhabditis briggsae*. Information in this area is primarily accessible through the main web portal http://www.wormbase.org. Our sister portal, http://parasite.wormbase.org, serves genomic information for over 100 nematodes and 30 platyhelminthes. More recently, we joined the Alliance of Genome Resources (AGR) to provide a uniform portal of knowledge for major model organisms for medical research. AGR members include the Mouse Genome Database (MGD), Rat Genome Database (RGD), Zebrafish Model Organism Database (ZFIN), FlyBase (FB), Saccharomyces Genome Database (SGD) and the Gene Ontology (GO).

Our goal is to facilitate research by providing up-to-date, high quality and curated information that is useful, easy to find and, easy to comprehend. To this end, we constantly make improvements to data acquisition, processing and presentation. Recent advances in experimental technology such as rapid whole genome sequencing and genome editing, coupled with stagnant funding, have further motivated WormBase to improve efficiency. Here we report our recent and significant progress.

## DATA ACQUISITION AND CURATION

### Community curation of research literature

**Figure 1.**
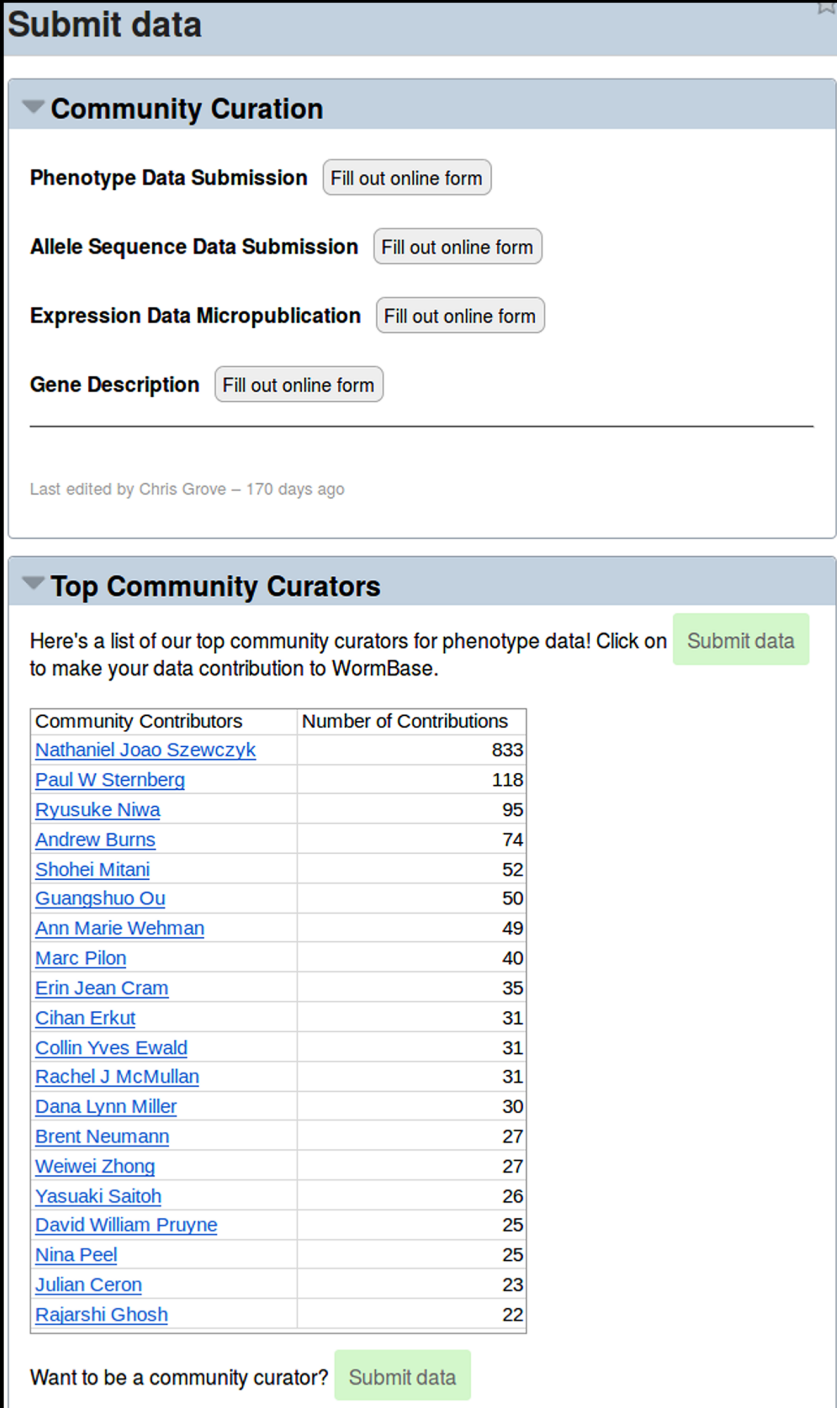
WormBase portal for community curation submission. On the data submission portal page, the top widget has links to four different forms for community curation data entry; the lower widget shows top contributors of community curation.

**Figure 2.**
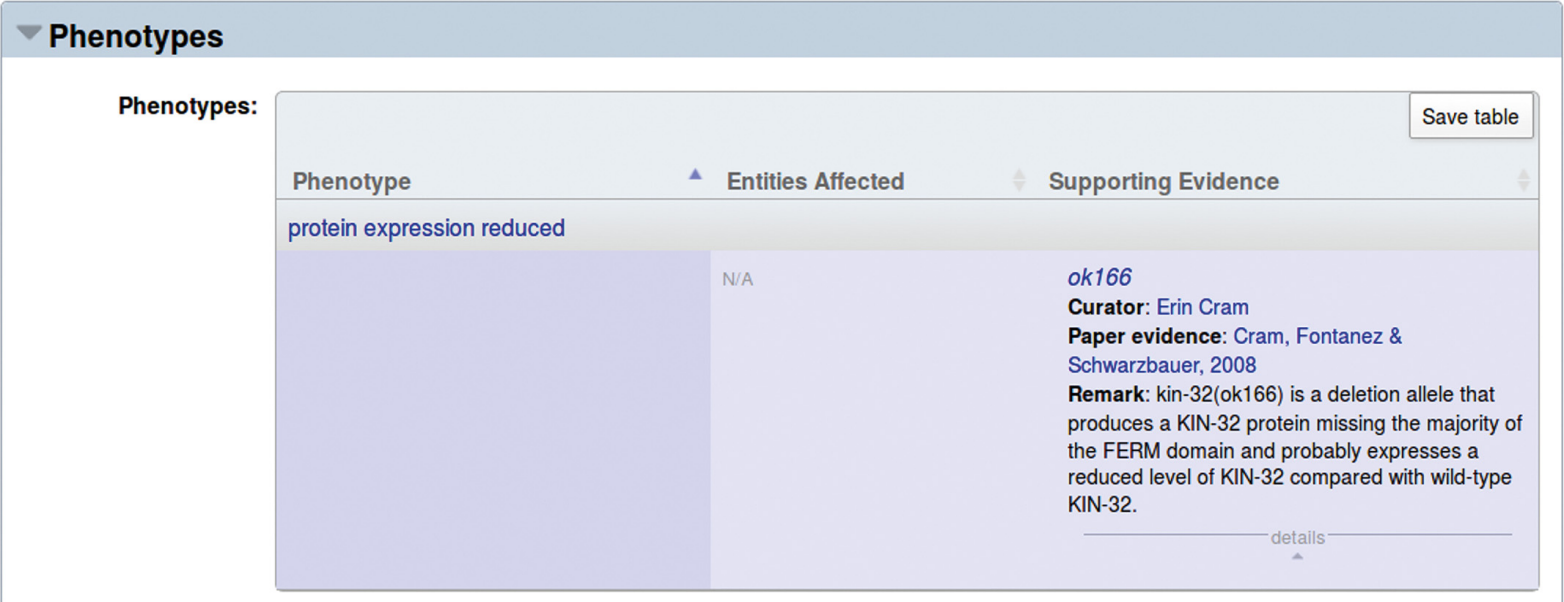
Phenotype widget of WormBase *kin-22* gene report page. In the phenotype widget on the *kin-22* gene page, the annotation ‘protein expression reduced’ phenotype caused by the *ok166* allele, is attributed to a community curator Erin Cram.

As WormBase strives to remain up-to-date with the curation of many data types from the literature, the number of new publications each year precludes comprehensive curation by WormBase curators alone. WormBase curators curate an average of about 120 papers per year per data type, yet the research literature is expanding on average by about 250 papers per year per data type. We have thus created a number of simple-to-use forms available on the WormBase website to facilitate community curation of several data types (Figure 1). These forms are to capture (i) published phenotypes resulting from alleles, RNAi knockdown and transgenic overexpression, (ii) allele sequence information and (iii) gene summaries to be displayed in the Overview widget of each WormBase gene page. In the past 2 years, we have piloted an outreach pipeline whereby authors of papers are sent an email requesting their curation of phenotype data from their paper. WormBase has sent 2282 emails (one solicitation email per paper in need of phenotype curation) and has received community curation for 362 of these papers (16% response rate). We have also received community curation annotations for papers for which we did not send a data curation request. In total, as of August 2017, WormBase has received 3337 annotations for 484 papers from 325 unique community curators. We track the contributions from each community curator and display the top 20 contributors on our ‘Submit Data’ page (Figure 1) as well as acknowledge individual community curators on the web pages for the data submitted (Figure 2). Encouraged by these initial results, WormBase will continue requesting phenotype curation from the community and will work on developing additional outreach pipelines and forms for community curation.

### Micropublication

**Figure 3.**
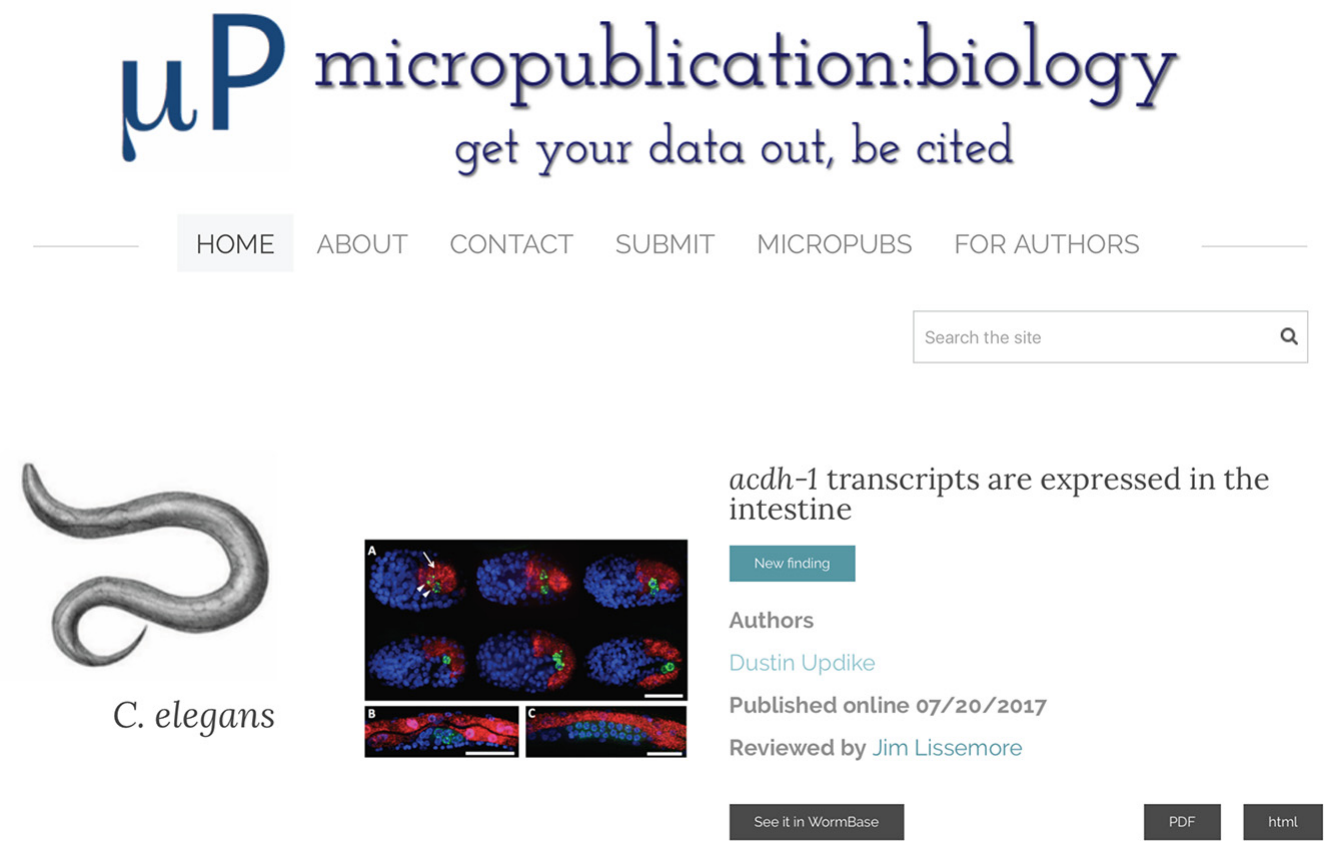
Homepage of the *Micropublication:biology* platform at http://www.micropublication.org.

WormBase also encourages the nematode research community to submit their unpublished research findings, in particular, data that traditionally remain unpublished for various reasons: space limitations imposed by science journals, project limitations due to funding, omission in narratives due to publication bias, e.g. negative results, or results that are not groundbreaking. Over the last couple of years WormBase has been developing a new publication venue, the recently launched *Micropublication:biology* (www.micropublication.org), for capturing these ‘orphan’ data (Figure 3). While in the past these submissions were captured in our database as ‘Personal Communications’, users will now be encouraged to submit their data through this formal scientific publication pipeline to ensure that they get trackable credit for their work. The new submission platform guides authors through datatype-specific online forms or templates that adhere to WormBase established nomenclature and data standards, which ensure these data are easily incorporated into the database. Further, the submission is peer-reviewed for clarity in result reporting and experiment reproducibility. Each publication is assigned a unique digital object identifier (DOI), providing a universal, persistent and citable reference. The end result is that research data are quickly published online in *Micropublication:biology* and discoverable in WormBase.

*Micropublication:biology* currently handles gene expression data and phenotype data, which can be submitted here: http://www.micropublication.org/submit. Over the next year, improvements will be made to existing forms and new forms will be added to cover additional data types, such as gene regulation, protein–protein/nucleic acid interactions, and human disease models. This platform will also be expanding to the greater AGR communities and databases.

### Curation of *C. elegans* models of human disease

WormBase curates *C. elegans* models of human disease. Genes are flagged as ‘Experimental models’ based on manual curation from the *C. elegans* literature or as ‘Potential models’ based on orthology to human genes from Ensembl through the Ensembl-Compara (1) pipeline and subsequent mapping to the genes implicated in disease from the OMIM (Online Mendelian Inheritance in Man) catalog (2). These genes that are curated as potential and/or experimental models are associated with human diseases via Disease Ontology terms from the Disease Ontology (3). To date, over 300 genes have been curated as experimental models and over a 1000 genes have been curated as potential models. WormBase together with other members of the AGR have been working to standardize and integrate disease data into common formats, towards the purpose of having unified displays of this data.

### Reannotation of the *C. briggsae* genome

WormBase resources now contain data on nearly 100 nematode species in addition to *C. elegans*. For a small selection of ‘core’ species with high quality reference genomes and established research interest (http://www.wormbase.org/species), we act as custodians of the genome sequence and annotation, curating gene structures and other sequence features, assigning and tracking identifiers and maintaining a complete historical record of all updates. Outside of these ‘core’ species, we import and display genomes and annotations provided by the nematode research community.

*C. briggsae* was the second nematode to have its genome sequenced, and has been a core species in WormBase since the initial publication of the genome (4). We have worked with the community to adopt and integrate new assemblies as they become available (5), and have manually curated gene structures by using genomic alignments of high-quality data (6). We recently embarked on a global reannotation of the *C. briggsae* data using the latest transcriptome data.

We selected paired-end RNA-seq experiments from the Short-Read Archive and processed them using the Trinity package (7) to produce a set of around 60 000 assembled transcripts. A number of triage, QA/QC and post-processing steps ultimately gave rise to a set of just over 21 000 putative, predicted coding DNA sequence (CDS) structures.

We then compared the predicted structures to the structures of the existing reference gene set. Approximately one third of the reference structures were matched by a predicted structure; about one third did not have significant overlap with any predicted structure; and the final third overlapped but disagreed with a predicted structure. These last third were reviewed manually by our curators. In around 6000 cases, the predicted structure was deemed to be correct and the reference structure updated accordingly; in ∼1000 cases, the reference structure was deemed to be correct; and in about 1000 cases, the locus was complicated and required extensive manual editing. Review/curation has just begun for the one third of the reference transcripts that do not have a significant overlap with a predicted structure.

The UTRs of coding transcripts are annotated in WormBase by comparing alignments of mRNAs and ESTs with the curated CDSs. *C. briggsae* has very few available mRNA and EST sequences and so the number of annotated UTR regions has previously been low, with only 2% of transcripts having an annotated 5′ or 3′ UTR, and 0.5% having both a 5′ and 3′ UTR. By now using the Trinity transcript alignments in this process, these proportions have increased significantly to (respectively) 87 and 54%.

## DATA QUERY TOOLS

### WormMine

WormBase’s primary data mining service is based on the open source data warehousing software platform InterMine (8). InterMine facilitates the integration and complex queries of related biological data in a powerful yet flexible web application. Since our last update, we have expanded the number of data types available to include RNA interference, gene classes, and strains. We have also made extensive improvements to preexisting data classes.

To make querying WormMine easier, we are updating and expanding ‘templates’, which are pre-canned queries for common datasets. These templates can be edited directly by users and may serve the additional purpose of introducing the underlying data model to the user. In forthcoming releases, a number of improvements will be available, including an expanded ontology mode that will add anatomy and phenotype terms, new WormBase data classes and a completely revamped web interface based on the InterMine’s new Bluegenes interface.

Of interest to readers who administrate InterMine instances, our InterMine infrastructure now runs entirely on the Amazon Elastic Compute Cloud (AWS EC2) with a fully automated deployment of new releases. In the past couple of years, our customized version of the database has undergone a complete code-base update and upgrade, at the same time that installation, data processing and testing procedures were streamlined.

### Gene set enrichment analysis

Genome-wide, large-scale experiments often generate a large number of gene sets that are correlated in a specific way, such as being co-localized, co-expressed or otherwise co-regulated. It can be time-consuming and difficult to manually find these correlations. We thus provide a gene set enrichment analysis (EA) tool (9, BioRxiv: https://doi.org/10.1101/106369) that compares the query gene set with existing annotated data of gene expression (with anatomy), gene function (with Gene Ontology) or phenotype to identify over-represented aspects. This tool is very easy to use: a user inputs a list of genes that may be obtained from RNA-seq analysis by comparing specific mutant and wild-type strains and the EA tool will return a list of significantly ‘enriched’ terms of anatomy, Gene Ontology and phenotype. Since WormBase is the source of the annotated data, an important feature of our tool is that it does not become stale; its underlying data are updated with each WormBase release.

### SimpleMine

SimpleMine is a bulk download tool that WormBase launched in the last year. Users can submit a list of gene names to get a tab-delimited file containing gene IDs from different naming sources, phenotypes from alleles and RNAi studies, anatomical features and developmental life stage of gene expression from individual and large-scale studies, human orthologs, human disease relevance as well as summarized descriptions about gene functions. SimpleMine can be accessed from the WormBase Tool menu. In contrast to WormMine, SimpleMine is designed to make common, basic queries as easy as possible, whereas WormMine allows the design of more sophisticated queries over a larger corpus of data.

## WEBSITE AND INFRASTRUCTURE

Since 2016, we have improved the overall organization and navigation of the website. Most notably, we created a new navigation menu styled as a drop down box that includes direct links to almost every feature available in WormBase, including resources, tools and user guides. This useful roadmap is available on every page across the site.

Many widgets and pages have been extended with new content and new visualization options. The Expression widget has been updated to include faceting of data by anatomy, life stage and localization. Similarly, the Fragments Per Kilobase of transcript per Million mapped reads (FPKM) expression figure of modENCODE data (10) has been streamlined. Users can now browse the curated intellectual relationships through a new visualization on the Person page (see below). The interaction viewer on Gene pages now allows better filtering based on types of interaction.

### Genome browsing

In the 2 years since WormBase’s new genome browser, JBrowse (11,12), was introduced, there have been several improvements both to the user interface and the data provided. Data from the *C. elegans* Natural Diversity Resource (CeNDR, 13) have been included as well as a ‘Landmarks’ track that mimics a similar track in the old genome browser (GBrowse, 14). Also, there are plans to incorporate more data tracks from external sources in the coming year, including sgRNA predictions to facilitate CRISPR experiments. JBrowse has a very flexible plugin architecture that makes extending its functionality possible in several ways. The JBrowse instance at WormBase takes advantage of that, both by using plugins created specifically for WormBase and by incorporating plugins written elsewhere. Plugins from outside sources include a tool to create high resolution screenshots (https://github.com/bhofmei/jbplugin-screenshot), to make turning on and off multiple tracks simultaneously easier (https://github.com/bhofmei/jbplugin-hierarchicalcheckbox) and to provide an interactive sequence viewer for gene and transcript features (https://github.com/tsaari88/FeatureSequence). Plugins that were written specifically for WormBase include a position weight matrix calculator and a tool to switch between the two track selection interfaces in JBrowse. Interested users can see all new JBrowse features at http://www.wormbase.org/tools/genome/jbrowse-simple/?data=data/c_elegans_PRJNA13758.

### SObA phenotype graph

WormBase has been annotating gene functions using the vocabulary of several ontologies. The more annotations there are for a given gene, the more difficult it becomes for users to comprehend the overall meaning of these gene functions. Are these functions related in some way? For example, the *C. elegans daf-16* gene, a forkhead transcription factor, has been annotated with 82 phenotype terms from a controlled vocabulary of 2400. Likewise, *daf-16* is annotated with 61 Gene Ontology terms, from over 40 000 total. Simply presenting these annotations as lists and tables is not particularly helpful. Therefore, we sought to remedy this problem with annotation graphs that are subgraphs of the full ontology, constructed with inference compilation and data-driven trimming.

We have implemented SObA (Summary of Ontology-based Annotations) graphs for gene phenotype annotations. Each SObA graph compiles all relevant phenotypes of the subject gene, directly annotated or inferred and the relationships among them; simplifies the graph to retain only the most informative phenotypes; and represents, by varying node sizes, the reproducibility and perhaps importance of each phenotype by the number of independent annotations it carries. SObA graphs are implemented using the Cytoscape javascript library (http://js.cytoscape.org/). Thus they support local zooming, highlighting and other controls. A user can readily traverse between a broad overview and a detailed inspection. For example, the *daf-16* graph is accessible here (http://www.wormbase.org/species/c_elegans/gene/WBGene00000912#b-c-10).

### Person intellectual lineage browser

The *C. elegans* research community is well known for its tight-knit personal and intellectual relationships and the spirit of collaboration. WormBase has been collecting and curating the intellectual lineage information of worm researchers for years and we now have a graph viewer to display it. The intellectual lineage graph is in a widget implemented on each Person page with two views. Using the simpler, direct view, one can see all the direct mentor and mentee relationships, centered on the focus person. One can then expand to the full lineage view, which additionally includes indirect, transitive relationships, such as the mentor of a mentor. An example may be found here (http://www.wormbase.org/resources/person/WBPerson42#2-10).

### WormBase ParaSite

WormBase ParaSite is a sub-portal of WormBase initially created to host the genomes of plant and animal parasitic nematodes and platyhelminths (15). It has now expanded to include almost all nematode and platyhelminth genomes, thus providing an integrated resource for exploring the genome biology of helminths in the evolutionary context of their free-living relatives. We periodically query GenBank/ENA/DDBJ and integrate new assemblies for which we can obtain primary annotation (protein-coding gene models), either from the sequence records or from the authors directly. In the past 2 years, the number of genomes included in the resource has increased from 99 to 134. Additions include *Globodera rostochiensis*, a plant parasitic nematode (16); and 16 genomes of the *Trichinella* complex (17), causative agents of the food-borne disease trichinellosis. We have also added a number of additional tools to the portal, including graphical displays of genome assembly quality and gene expression tracks on the genome browser. One notable recent update is the addition of a JBrowse browser for every genome, which includes all tracks from the integrated Ensembl-based browser. JBrowse genome browsing is thus now available for the entire set of nematode and flatworm genomes across WormBase and WormBase ParaSite.

### Infrastructure

The system architecture of WormBase comprises a heterogeneous collection of different database technologies, including traditional relational databases, modern document stores and the biological object/graph database AceDB. AceDB has been a central tool for WormBase since its inception, but it does not scale naturally to modern large-scale genomics data, and support for it has now officially ceased. We therefore began a project a few years ago to migrate our service and curation workflows away from AceDB.

The new system architecture we are working towards makes heavy use of Datomic (http://www.datomic.com), a graph-oriented database management system that has built-in support for transactions, point-in-time querying and full audit trail for every update operation. By moving to a modern database, we aim to open new opportunities for curation, to reduce the complexity of our build and hosting processes and increase the reliability of our services.

Currently, we are maintaining a hybrid system split between AceDB and Datomic as we procedurally port functionality of the website to Datomic. This strategy has allowed us to continue presenting new data and features with no downtime to end users. About half of the current www.wormbase.org website is now served on the fly from our Datomic architecture.

Although there have been minor changes to our templating engine, we have almost completely rewritten our RESTful API. The API is now written in Clojure, with REST endpoints served via Swagger. Interested users can explore these endpoints at http://rest.wormbase.org/.

Throughout the process, we have also taken the opportunity to take advantage of modern deployment technologies. Our stack includes Amazon’s DynamoDB for storing the data, CloudWatch for monitoring, ElasticBeanstalk for deployment and Docker for enabling continuous integration. Using these technologies has allowed us to provide a robust and automated architecture that facilitates continuous deployment to production.

Also, we are improving search capabilities and adding new avenues for exploring data at WormBase. Previously, we used the Xapian indexer (https://xapian.org/) to drive the global text search at WormBase. This has now been rewritten using Elasticsearch (https://www.elastic.co), which offers improved relevance and ranking of results and greater speed. We continue to make WormBase data available programmatically through a number of RESTful APIs. API endpoints are documented in the Swagger specification (https://swagger.io/specification/) and allow users to explore them through a web interface (http://rest.wormbase.org/). We are also developing a new GraphQL Web API (http://graphql-dev.wormbase.org/), that features greater flexibility for accessing data via the GraphQL query language.

## THE ALLIANCE OF GENOME RESOURCES

WormBase has joined together with five other Model Organism Databases (MODs) and the Gene Ontology (GO) project to form the AGR. The AGR is working to develop a comprehensive knowledge portal in support of basic and translational research that will offer well-integrated information about model organism genetics and its relationship to human disease and biology. The AGR will serve a number of specific communities, including human geneticists and clinical researchers; basic science researchers; computational biologists and data scientists; and educators and students.

The formation of AGR builds on the collaborative activities between the MODs and GO over several years seeking to streamline data integration and exchange via the development and use of common data standards. The member groups will now merge key activities and data representations, coordinating data curation and integration within a common framework. For example, one of the initial targets for AGR is to define and adopt a common approach for the inference of orthology between model organisms and human genes. With the release of the first version of the AGR web portal in October 2017 (http://www.alliancegenome.org), users will be able to review core information about genes in human and the major model organisms, including descriptions of function and associations with human disease. As the integration of the MODs and GO teams progresses, a longer term aim is for AGR to become a framework for the integration of a wide variety of model organism and bioinformatics resources under a common platform, greatly facilitating data analysis and discovery.
